# An additional lysis procedure during arthrocentesis of the temporomandibular joint

**DOI:** 10.1186/s40902-021-00324-4

**Published:** 2021-10-12

**Authors:** Keon-Mo Lee, Wan-Hee Jang, Myoung-Sang You, Bu-Kyu Lee

**Affiliations:** 1 Department of Oral and Maxillofacial Surgery, Asan Institute for Life Sciences, Asan Medical Center, 88 Olympic-ro, 43-gil, Songpa-gu, Seoul, 05505 Republic of Korea; 2 College of Medicine, University of Ulsan, Seoul, Republic of Korea

**Keywords:** Temporomandibular joint, Temporomandibular joint disorder, Arthrocentesis, Needle detachment, Lysis of adhesion

## Abstract

**Background:**

Arthrocentesis of the temporomandibular joint (TMJ) is an easy, highly efficient, minimally invasive procedure for treating temporomandibular joint disorders (TMDs). However, in some cases of mouth opening limitation (MOL), routine arthrocentesis is ineffective due to severe fibrotic adhesion in the superior joint space of the TMJ. In this condition, mechanical lysis of the adhesions might be needed to resolve the MOL, as well as other symptoms, such as chronic pain. Currently, this can be achieved by arthroscopic surgery or open TMJ surgery. The objective of this study was to introduce and evaluate our trial of the adhesion lysis procedure during arthrocentesis of the TMJ using normal 18-gauge needles.

**Results:**

In this study, 40 patients with MOL due to disc derangement underwent conventional arthrocentesis at first and then physical detachment was conducted using the same needle. The change in maximum mouth opening (MMO) and the pain at the TMJ were recorded before, during, and after treatment according to our protocol. The mean increase in MMO after conventional arthrocentesis was 6.6 ± 4.2mm. The mean increase in MMO after the detachment procedure with the same needle was 4.2 ± 2.0 mm. The MMO in ten patients was significantly increased after the detachment procedure than after arthrocentesis alone. In all cases, the pain intensity in the TMJ significantly decreased over time, whereas the MMO increased over time. No adverse effect was observed in all joints during our observation periods.

**Conclusion:**

We confirmed that our simple lysis procedure with the same needle of the arthrocentesis of the TMJ could not only improve the MMO more than after a conventional arthrocentesis but also resolve severe adhesion of the joint space that was ineffective by conventional arthrocentesis. Although this additional lysis procedure is simple, it might reduce the number of cases of more invasive procedures such as arthroscopic surgery or open TMJ surgery.

## Background

The progression of temporomandibular disorders (TMDs) includes chronic inflammation of the joint that results in joint space adhesion to varying degrees. This could limit the natural movement of the condylar head and TMJ disc owing to increased friction in the joint space, which further aggravates disc displacement and inflammation of the TMJ. To relieve this friction and inflammation of the joint space, arthrocentesis was introduced in 1999 [1]. Since then, arthrocentesis of the TMJ has proved to be an easy, highly efficient, minimally invasive procedure for TMDs and it is now regarded as an essential option for treating various advanced-stage TMDs with internal derangement [2, 3]. To date, several indications for arthrocentesis (such as MOL) have been proposed [4, 5]. With these indications, the success rate of arthrocentesis of the TMJ has been reported to be as high as 85–95% [6]. However, in cases of severe joint space adhesion, arthrocentesis is often ineffective because its lysis power mainly depends on hydraulic pressure through a needle with manual pumping action; thus, it is too weak to detach severe adhesions in the joint space. In such cases, arthroscopic surgery or open TMJ surgery is opted for because those two procedures provide more power to detach existing adhesions in the joint space. However, arthroscopic surgery and open TMJ surgery are quite demanding physically and economically for both patients and physicians. Those procedures are conducted under general anesthesia and have more potential postoperative complications, such as visible scars and aggravation of symptoms. In addition, the more lysis of the joint space having adhesions, the better movement of the mandibular condyle.

Therefore, to improve the release of superior joint adhesions in the TMJ in an easier way, we devised a technique of swiping the needle in the joint space using the same needle during routine arthrocentesis. In this study, we evaluated the clinical feasibility of this technique.

## Methods

**Fig. 1:**
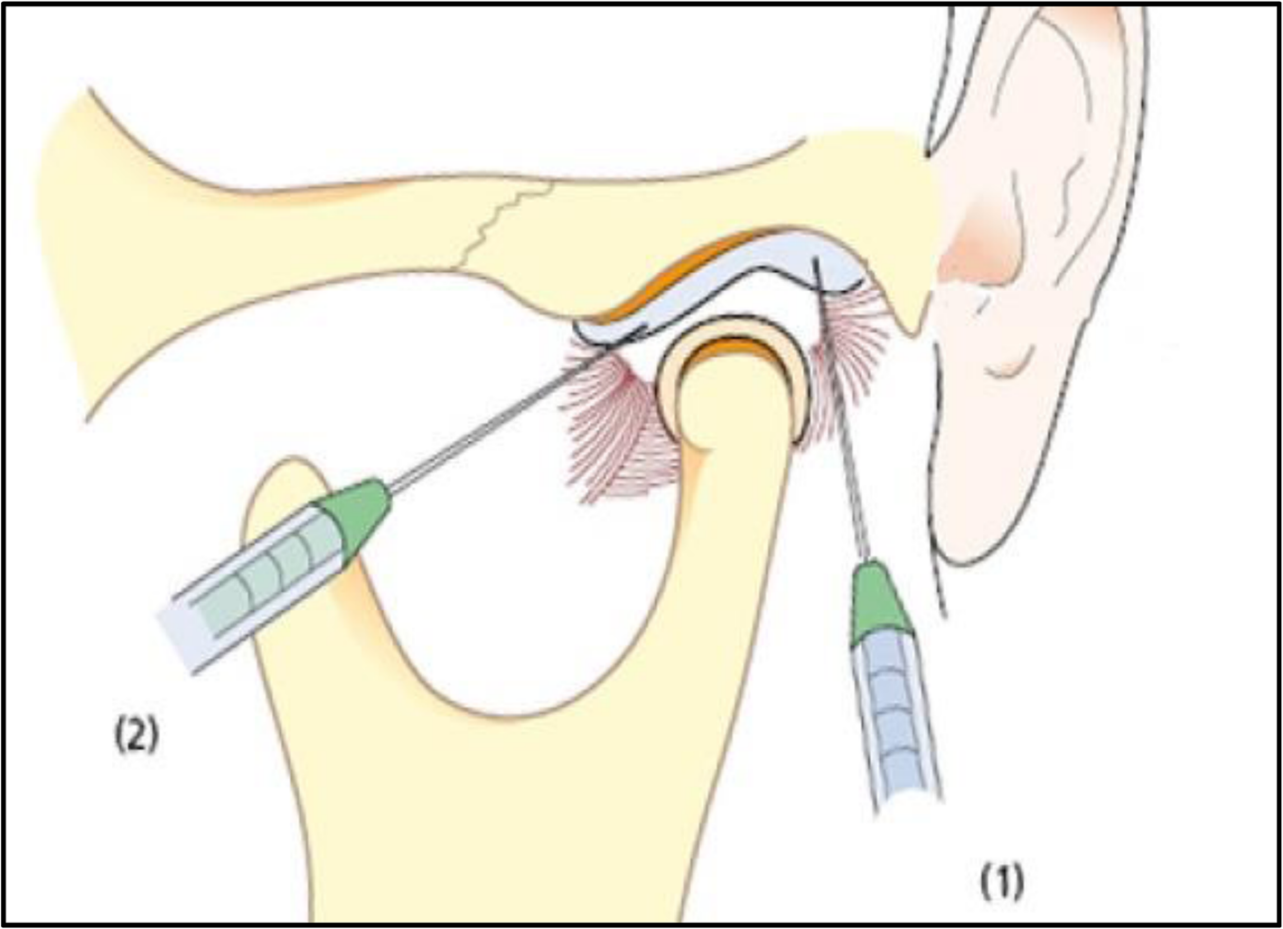
Schematic of the location of the two needles for arthrocentesis and detachment procedure

**Fig. 2:**
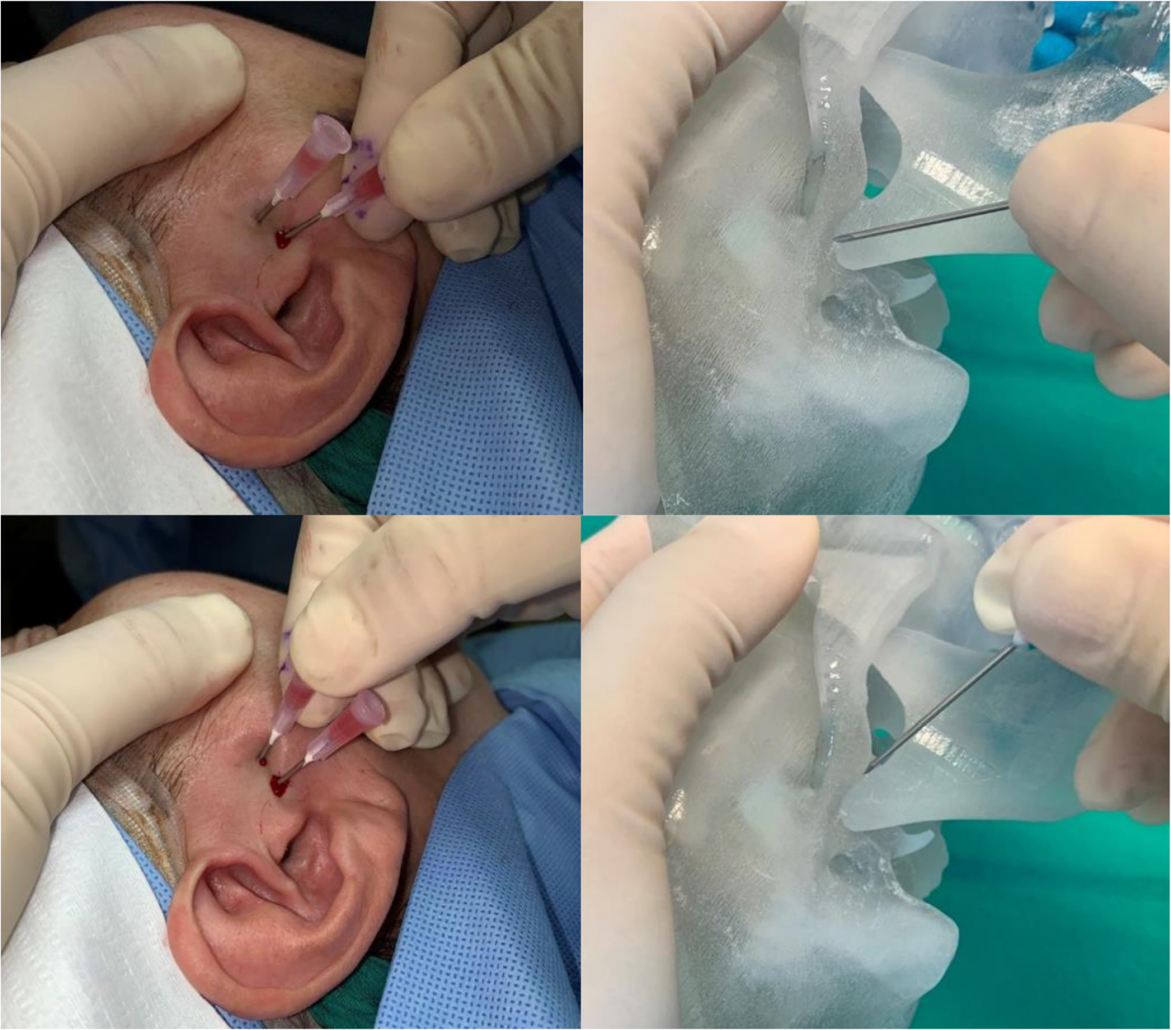
Surgical procedure used for patients illustrated with the rapid prototyping model

A total of 40 patients were enrolled. They were adult females aged from 20 to 68 years (mean age, 46.6 ± 13.6years), complaining of MOL and disc displacement without reduction confirmed using magnetic resonance imaging (MRI). They had no history of previous TMJ surgery and had received routine conservative treatments such as stabilization using splints and physical therapy for 3 months before the surgical procedure. The patients underwent routine arthrocentesis and our detachment procedure using 18-gauge needles in the same way performed by the same surgeon. Following treatment, patients were asked to perform the mouth opening exercise (MOE) using their own fingers as wide as possible from the day after the surgical procedure and to follow our routine postoperative management protocol (such as soft diet) for 4 weeks, immediate splint wearing, and taking analgesics for a week. Patients were examined clinically and radiologically prior to and after the procedure. According to MRI, the patients who were diagnosed with TMJ disc displacement without reduction were included in this study. MOL without disc derangement was excluded from this study to decrease potential variables in determining the efficacy of arthrocentesis and needle detachment procedure. Our procedures are illustrated in Figs. 1 and 2. This procedure was performed under local anesthesia without intravenous sedation because the patient’s cooperation in a clear conscious state was necessary when measuring the MMO.

Briefly, after marking the position for needle insertion, an 18-gauge needle was carefully inserted into the superior joint space of the TMJ. Approximately 2 mL of saline was injected, and reflux of the saline was confirmed by several pumping actions, which is critical in determining if the needle tip is at the right position in the superior joint space. After routine arthrocentesis, the MMO, which is the distance between the edges of the central incisors of the maxilla and the mandible, was measured. The detachment procedure was then performed with the same needle at the fossa and posterior part of the eminence, and the value was recorded. Then, an additional needle was inserted at the ridge of the articular eminence area and swiped to detach anterior adhesions in the joint. After that, the MMO was measured and recorded. At the end of the procedure, a commercial anti-adhesive agent containing hyaluronic acid (Gardix®, Hanmi Pharmacy; Seoul, Korea) and dexamethasone (Yuhan Pharmacy; Seoul, Korea) was injected in both TMJs. The patient took routine antibiotics and analgesics for 3 days and MOE using both hands, starting at 6 times a set and 6 sets a day from the day after the surgical intervention. All conservative treatments, such as splint wearing, massage, and careful chewing, were continued throughout the follow-up period. Any complications during or after surgery were also recorded to evaluate the safety of this procedure. A paired *t* test was used to evaluate postoperative changes of MMO. Probability values less than 0.05 were deemed statistically significant. SPSS Statistics version 25.0 software (SPSS Inc., Chicago, IL, USA) was used for all statistical analysis.

## Results

The mean preoperative MMO was 33.3 ± 3.7 mm. As a result of arthrocentesis using an 18-gauge needle enabled the instant increase of mouth opening, with mean MMO of 39.9 ± 3.7 mm.

**Fig. 3:**
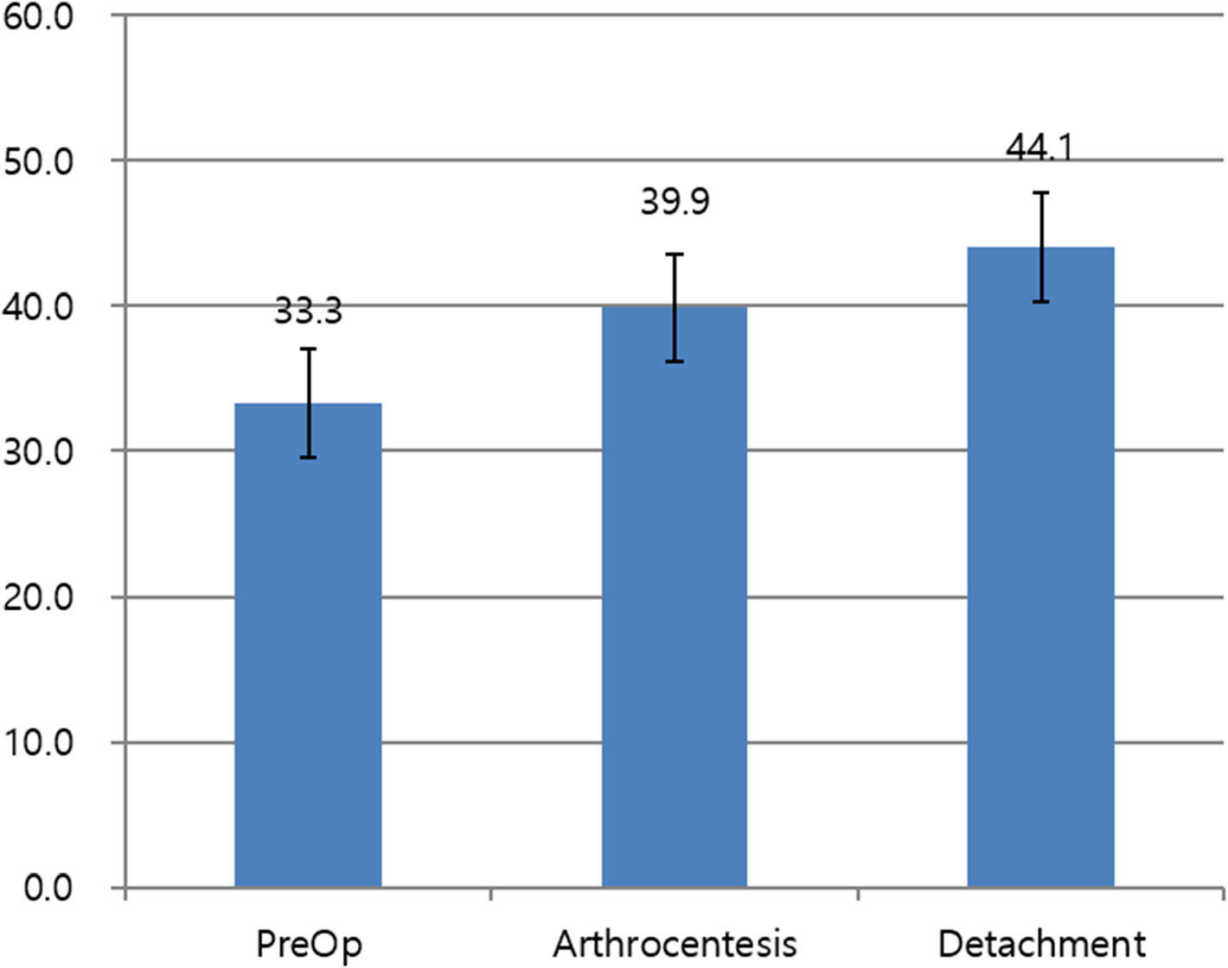
Mean maximum mouth opening (preoperative, after arthrocentesis, after additional detachment procedure)

The mean increase in MMO after arthrocentesis was 6.6 ± 4.2 mm. After the additional detachment procedure, the mean MMO was 44.1 ± 3.7 mm. The mean increase in MMO after detachment was 4.2 ± 2.0 mm (Fig. 3). This is an additional MMO increase by the detachment procedure. There were some dramatic changes in some patients through this detachment procedure. In 10 cases, the MMO increase obtained by detachment was larger than obtained by arthrocentesis. Even for six cases of these, the difference was more than double.

These examples suggest that this simple detachment procedure could not only further increase the MMO but also resolve severe adhesion in the joint space without the need for more serious procedures such as arthroscopic or open TMJ surgeries. Furthermore, in some cases, with this simple detachment procedure, the displaced disc position was slightly restored to a less displaced position. However, in most cases, disc positions were unchanged. No complications regarding the detachment procedure were observed in any case.

**Fig. 4:**
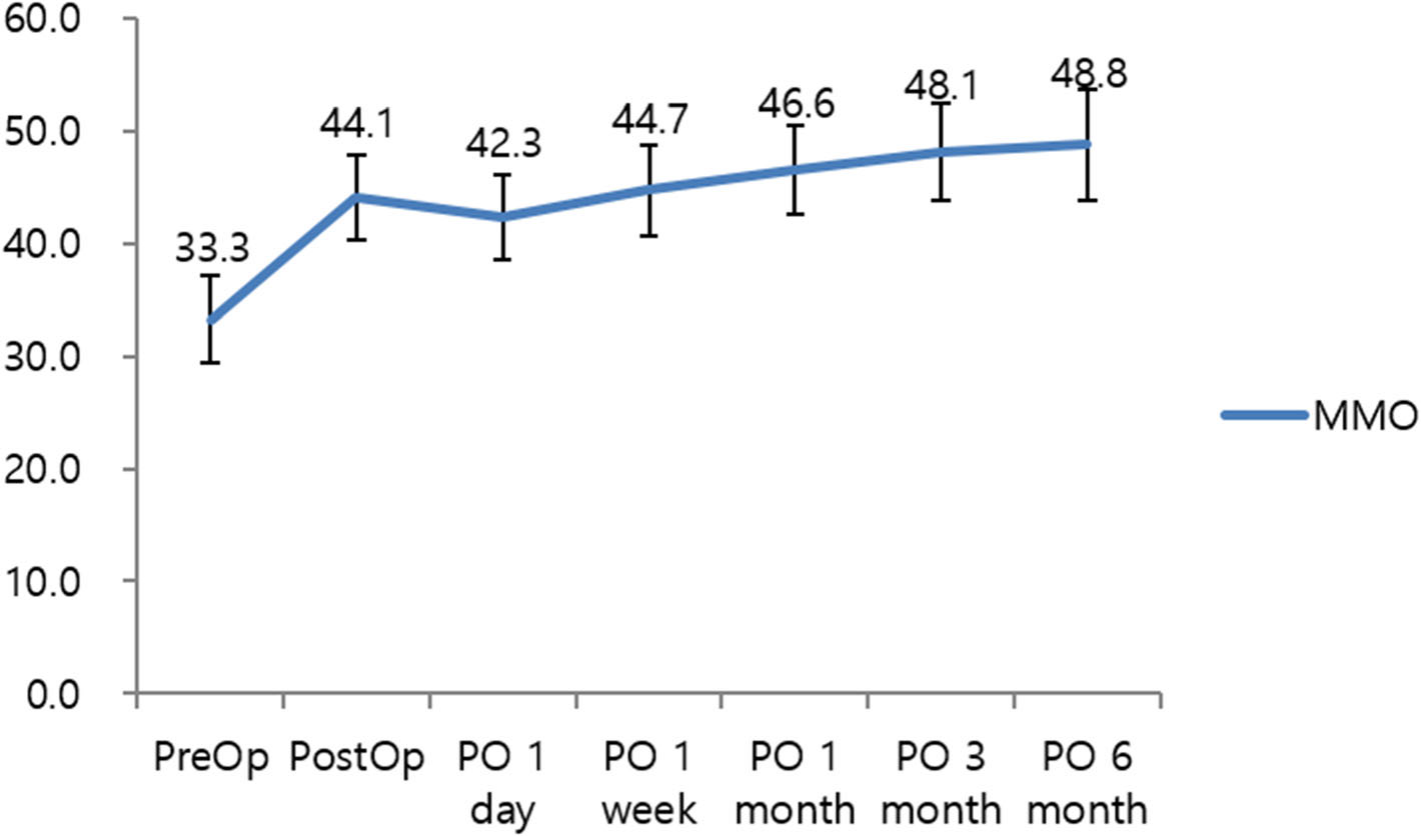
Maximum mouth opening after surgery and during follow-up visits

**Fig. 5:**
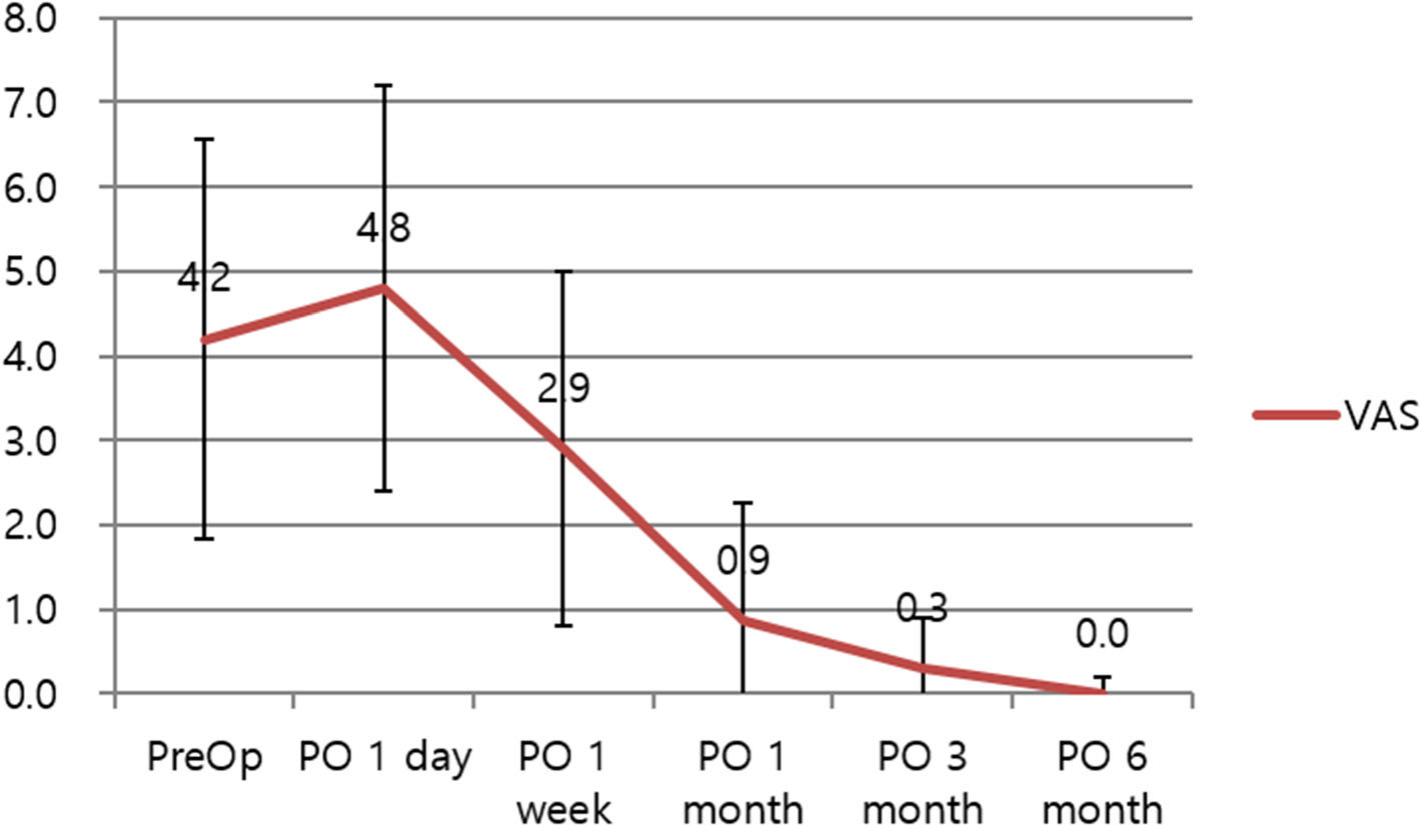
Visual analog scale (VAS) score after surgery and during follow-up visits

The MMO was maintained postoperatively and gradually increased during the observation period. The MMO and visual analog scale (VAS) score measured during the follow-up period (6 months) are shown in Figs. 4 and 5. The day after surgery, the MMO decreased due to acute pain in the TMJ induced by surgical trauma. The detachment procedure using two needles did not cause as severe and persistent pain as general arthrocentesis procedures.

## Discussion

Nowadays, arthrocentesis of the TMJ has proven to be an essential option to reduce joint pain, improve mouth opening, and reduce clicking [7–10]. It is most commonly used to treat patients with anterior disc displacement without reduction (closed lock) and disc adhesion to the adjacent joint structure [7–10]. Given that arthrocentesis can have a similar clinical outcome and can be performed under local anesthesia, it is more beneficial for both clinicians and patients. However, in cases of severe adhesion in the TMJ, arthrocentesis is even ineffective because the pumping power generated by arthrocentesis is not enough to lyse adhesions. Furthermore, some of the cases of MOL recurred after arthrocentesis due to residual adhesion of the joint space even after arthrocentesis. Therefore, mechanical lysis of adhesions and lavage of the joint space of the TMJ are critical for those cases [11–13].

Lysis and lavage of the TMJ were first performed using arthroscopy by Ohnishi [14]; however, because it was found that visualizing the joint is not essential to accomplish these objectives and often leaves scars or perforations of the glenoid fossa, arthrocentesis became more popular as a substitute for TMJ arthroscopy [11].

As many previous studies and our results have shown, the MMO was instantly increased after arthrocentesis alone. However, similar to some cases in our study where the adhered joint space could not be effectively lysed using hydraulic pressure alone, our simple additional detachment procedure using the same needle and an additional needle significantly improved such conditions while still being minimally invasive. Furthermore, in cases of patients who achieved enough MMO gain by arthrocentesis alone, additional gains were observed following our detachment procedure. This finding implies that almost every joint space from disc displacement has some amount of adhesion that needs to be mechanically lysed. It is clear that this additional lysis of adhesions might be helpful in the delay or prevention of re-adhesion in the joint space afterward.

In addition, the major gains in MMO after our arthrocentesis and the detachment procedure in this study were mostly achieved by the first needle’s arthrocentesis and detachment action. Therefore, we assumed that the first needle plays a key role in the detachment of the joint space of the TMJ. This is supported by previous reports that adhesions of the TMJ disc were observed mainly at the articular fossa and eminence [15]. It is also because the vacuum in the joint cavity is resolved. With this in mind, for those who are still not good at placing multi-needles into the proper location of the joint space, the single-needle technique on the rationale that repeatedly pumping saline into the superior joint space of the TMJ could also be acceptable to get the acceptable clinical outcome to release joint adhesions [16]. It is also advantageous in that single-needle use carries fewer risks of infection, bleeding, and patient discomfort [17].

Although some early studies supported the efficacy of hyaluronic acid injections in treating internal TMJ derangements [18], more recent evidence suggested that it may be effective in inflammatory degenerative disorders as well, especially if combined with a thorough joint lavage [19, 20]. Such findings extended the indications for hyaluronic acid injections to a wider population of patients with TMD, especially in terms of age range, because a higher age of onset is recognized for inflammatory degenerative disorders with respect to other forms of TMD. In our study, hyaluronic acid injected immediately after arthrocentesis improved the signs and symptoms of patients with TMD.

Following the operation, continued care such as wearing an occlusal splint, performing mouth-opening exercises, and eating a soft diet, is also important. Considering the various etiologies of chronic TMD (stress, parafunctional habits, chronic tension of masticatory muscles, etc.), no surgical procedure can last forever, and the surgeon should keep in mind that only steady management of the patient’s education can provide a long-term effect from these surgical procedures.

In this study, no complications occurred during the detachment procedures. However, there might be potential contraindications when the cartilage layer is healthy. Articular cartilage lesions after trauma or damage in weight-bearing joints often fail to heal on their own and may be associated with pain, loss of function, and long-term complications such as osteoarthritis [21]. Osteochondral injuries are both naturally and therapeutically irreversible with current treatment parameters. Although inferior repair commonly occurs, stable regeneration of hyaline cartilage has never been documented [22]. Therefore, this procedure is contraindicated in adolescents in order to preserve the growing chondral layer in their articular cartilage.

For effective arthrocentesis of the TMJ, the needle is directed to the glenoid fossa. The glenoid fossa is thin (0.5–1.5 mm) [16] and could be rendered thinner by erosion owing to degenerative changes by chronic inflammation of the joint space. As the dura and temporal lobe of the brain are located just above the glenoid fossa, iatrogenic perforation of these structures could occur via either arthroscopy or arthrocentesis. Therefore, the surgeon should not penetrate that far. A penetration of approximately 25 mm is enough to reach the upper joint space [7]. Technically, perforation can be avoided with information regarding the status of the articular fossa by physically feeling the pressure on the inserted needle and using MRI prior to the procedure.

Causes of MOL include disc displacement, facial trauma, chronic tension of the masticatory muscles, and tumors in the TMJ area [23]. In our MRI findings, in several cases, chronic MOL persisted without disc displacement and did not improve with either medication or conservative treatment. These cases could be resolved by mechanical detachment of the joints and were diagnosed as stuck discs of the TMJ. In addition, although we excluded it from our study, there was a case of MOL caused by neurological factors. Therefore, determining the cause of MOL before performing the procedure on a patient is critical.

## Conclusion

Our detachment technique with 18-gauge needles was helpful in treating patients with adhesions in the TMJ. Additional detachment of fibrotic adhesions within the joint space of the TMJ using needles during routine arthrocentesis of the TMJ could achieve a higher increase in the MMO than that of arthrocentesis alone. Furthermore, the detachment procedure could resolve cases with severe fibrotic adhesions in the TMJ that cannot be resolved using routine arthrocentesis of the TMJ. There was no complication during and after this procedure. With these results, we can conclude that our detachment procedure is an effective and safe option to complement conventional arthrocentesis.

## Data Availability

Not applicable

## Abbreviations

TMJ: Temporomandibular joint
TMDs: Temporomandibular joint disorders
MOL: Mouth opening limitation
MRI: Magnetic resonance imaging
MOE: Mouth opening exercise
VAS: Visual analog scale

## References

[1] Frost DE, Kendell BD (1999). Part II: The use of arthrocentesis for treatment of temporomandibular joint disorders. J Oral Maxillofac Surg.

[2] Barkin S, Weinberg S (2000). Internal derangements of the temporomandibular joint: the role of arthroscopic surgery and arthrocentesis. J Can Dent Assoc.

[3] Yi AN, Han SY, Yun KI (2000). Clinical aspect of arthrocentesis. J Korean Assoc Oral Maxillofac Surg.

[4] Gonzalez-Garcia R (2015). The current role and the future of minimally invasive temporomandibular joint surgery. Oral Maxillofac Surg Clin North Am.

[5] Wilkes CH (1989). Internal derangements of the temporomandibular joint: pathological variations. Arch Otolaryngol Head Neck Surg.

[6] Nitzan DW, Samson B, Better H (1997). Long-term outcome of arthrocentesis for sudden-onset, persistent, severe closed lock of the temporomandibular joint. J Oral Maxillofac Surg.

[7] Tozoglu S, Al-Belasy FA, Dolwick MF (2011). A review of techniques of lysis and lavage of the TMJ. Br J Oral Maxillofac Surg.

[8] Nitzan DW, Dolwick MF, Heft MW (1990). Arthroscopic lavage and lysis of the temporomandibular joint: a change in perspective. J Oral Maxillofac Surg.

[9] Nitzan DW, Dolwick MF, Martinez GA (1991). Temporomandibular joint arthrocentesis: a simplified treatment for severe, limited mouth opening. J Oral Maxillofac Surg.

[10] Kim CW, Lee SJ, Kim EH, Lee DK, Kang MH, Song IS, Jun SH (2019). Effect of arthrocentesis on the clinical outcome of various treatment methods for temporomandibular joint disorders. Maxillofac Plast Reconstr Surg.

[11] Nitzan DW (2006). Arthrocentesis--incentives for using this minimally invasive approach for temporomandibular disorders. Oral Maxillofac Surg Clin North Am.

[12] Dolwick MF (2007) Temporomandibular joint surgery for internal derangement. Dent Clin North Am 51:195-208, vii-viii. 10.1016/j.cden.2006.10.003 17185066

[13] Kim CH, Hwang HS, Sin SH (2003). The study of the predictors in arthrocentesis and lavage of temporomandibular joint disorder: retrospective evaluation of anterior disc displacement without reduction. J Korean Assoc Oral Maxillofac Surg.

[14] Ohnishi M (1990). Arthroscopy and arthroscopic surgery of the temporomandibular joint (T.M.J.). Rev Stomatol Chir Maxillofac.

[15] Warnke T, Carls FR, Sailer HF (1996). A new method for assessing the temporomandibular joint quantitatively by dental scan. J Craniomaxillofac Surg.

[16] Guarda-Nardini L, Ferronato G, Manfredini D (2012). Two-needle vs. single-needle technique for TMJ arthrocentesis plus hyaluronic acid injections: a comparative trial over a six-month follow up. Int J Oral Maxillofac Surg.

[17] Carroll TA, Smith K, Jakubowski J (2000). Extradural haematoma following temporomandibular joint arthrocentesis and lavage. Br J Neurosurg.

[18] Kim JJ (2006). The effect of intra-articular injection of hyaluronic acid after arthrocentesis in treatment of internal derangements of the TMJ. J Korean Assoc Oral Maxillofac Surg.

[19] Guarda-Nardini L, Olivo M, Ferronato G, Salmaso L, Bonnini S, Manfredini D (2012). Treatment effectiveness of arthrocentesis plus hyaluronic acid injections in different age groups of patients with temporomandibular joint osteoarthritis. J Oral Maxillofac Surg.

[20] Alpaslan GH, Alpaslan C (2001). Efficacy of temporomandibular joint arthrocentesis with and without injection of sodium hyaluronate in treatment of internal derangements. J Oral Maxillofac Surg.

[21] Falah M, Nierenberg G, Soudry M, Hayden M, Volpin G (2010). Treatment of articular cartilage lesions of the knee. Int Orthop.

[22] Willers C, Wood DJ, Zheng MH (2003). A current review on the biology and treatment of articular cartilage defects (Part I & Part II). J Musculoskelet Res.

[23] Yun PY, Kim YK (2005). The role of facial trauma as a possible etiologic factor in temporomandibular joint disorder. J Oral Maxillofac Surg.

